# Control of Ion Transport by Tmem16a Expressed in Murine Intestine

**DOI:** 10.3389/fphys.2019.01262

**Published:** 2019-10-04

**Authors:** Karl Kunzelmann, Raquel Centeio, Podchanart Wanitchakool, Inês Cabrita, Roberta Benedetto, Tultul Saha, Kazi Mirajul Hoque, Rainer Schreiber

**Affiliations:** ^1^Institut für Physiologie, Universität Regensburg, Regensburg, Germany; ^2^Division of Pathophysiology, National Institute of Cholera and Enteric Diseases, Kolkata, India; ^3^Department of Physiology, University of Maryland School of Medicine, Baltimore, MD, United States

**Keywords:** Tmem16a, anoctamin 1, Cl^–^ secretion, mucus secretion, cystic fibrosis, Ca^2+^ signaling

## Abstract

Cl^–^ secretion by the human and murine intestinal epithelium occurs through the cystic fibrosis transmembrane conductance regulator (cftr). However, the Ca^2+^ activated Cl^–^ channel Tmem16a was shown to contribute to Cl^–^ secretion, mainly, but not exclusively, as a basolaterally located Cl^–^ channel that controls basolateral Ca^2+^ signaling, and thus activation of basolateral Ca^2+^ dependent Sk4 K^+^ channels. In intestinal goblet cells, Tmem16a was shown to regulated Ca^2+^ signals required for exocytosis of mucus. Because a recent report denied the existence and functional role of Tmem16a in murine intestine, we reexamined in detail expression of mRNA and protein for Tmem16a in mouse colon. In experiments using short-circuited Ussing chamber and whole cell patch-clamp techniques, we further compared ion transport in wild type (WT) colon with that in mice with intestinal epithelial specific knockout of Tmem16a. As reported earlier we fully confirm expression of Tmem16a in colonic epithelial cells and the role of Tmem16a for both Ca^2+^-dependent and cAMP-regulated ion secretion.

## Introduction

Cl^–^ secretion by human and murine intestinal epithelium occurs through the cystic fibrosis transmembrane conductance regulator (cftr). We were the first showing that in normal adult human and murine colon, cftr is the only luminal Cl^–^ exit pathway (Mall et al., 1998; Schreiber et al., 2015). Yet, the situation might be somewhat different during intestinal disease, in young infants and in intestine epithelium of mouse pups. In large intestine of homozygous F508del-CFTR patients, we did not find evidence for a compensatory Ca^2+^ activated Cl^–^ channel (CaCC) (Hirtz et al., 2004). Moreover, DIDS, CFTR-inh172 and other Cl^–^ channel inhibitors cross-react with both types of Cl^–^ currents, and therefore cannot be used to dissect Ca^2+^ and cAMP-dependent Cl^–^ secretion (Lerias et al., 2018).

In our own previous studies (Ousingsawat et al., 2009; Schreiber et al., 2010; Ousingsawat et al., 2011; Kunzelmann and Schreiber, 2014; Schreiber et al., 2015; Benedetto et al., 2017; Benedetto et al., 2019a, b), and in reports by other teams (Gomez-Pinilla et al., 2009; He et al., 2011; Hoque et al., 2012; Mroz and Keely, 2012; Yin et al., 2017; Rottgen et al., 2018; Lee et al., 2019), expression of Tmem16a in murine intestine was convincingly demonstrated. A gradual decrease of expression from distal colon toward small intestine was shown previously (Wanitchakool et al., 2014).

Crucially, a defect in intestinal ion transport upon knockout of Tmem16a was shown in different knockout models, while a role of Tmem16a for intestinal ion transport has been reported by at least four different teams (Ousingsawat et al., 2009; He et al., 2011; Hoque et al., 2012; Benedetto et al., 2017; Lee et al., 2019). Despite this abundant evidence, a recent report by Vega et al. (2019) claimed that the Ca^2+^ activated Cl^–^ channel Tmem16a is not expressed in mouse intestinal epithelium. Moreover, Tmem16a was found to be irrelevant for Cl^–^ transport and mucus properties in mouse intestine, using mice with epithelial specific knockout of Tmem16a. These surprising results prompted us to reexamine expression of Tmem16a in murine intestine using various Tmem16a antibodies. We analyzed intestinal mucosa in short circuited Ussing chamber in the presence of bicarbonate/CO_2_ (25 mM/5%; CO_2_/bic) buffered solution, to compare Ca^2+^ and cAMP-dependent ion secretion in wild type (WT) mice and mice with intestinal epithelial knockout of Tmem16a (Tmem16a^fl/fl^Vil1Cre). The present data fully reconfirm our earlier findings and that of other teams.

## Materials and Methods

### Animals, Cells, Isolation of Crypts

All animal experiments were approved by the local ethics committee of the Government of Unterfranken/Würzburg (AZ: 55.2-2532-2-328) and were conducted according to the guidelines of the American Physiologic Society and the German law for the welfare of animals. Generation of Tmem16a^fl/fl^Vil1Cre mice and isolation of intestinal epithelial cells have been described earlier (Schreiber et al., 2015). Mice were anesthetized with CO_2_ and sacrificed by cervical dislocation. The large intestine was collected from the peritoneal cavity, washed and stored in ice-cold (Dulbecco’s PBS) DPBS. To isolate colonic epithelial cells the colon was everted and filled with Ca^2+^ free solution (in mM: NaCl 140; KCl 5; MgCl_2_^∗^6H_2_O 1; CaCl_2_^∗^2H_2_O 2; HEPES-Tris 10; D-Glucose 10). Following 20 min of incubation at 37°C the everted colonic sac was placed in high Ca^2+^ solution (in mM: NaCl 96, KCl 1,5; HEPES-Tris 10; Na-EDTA 27; Sorbitol 55; Sucrose 44) and the crypts obtained by mechanical shaking. All solutions were supplemented with 1 μM Indometacin and 10 μM DTT (both purchased from Sigma, Darmstadt, Germany). Cell pellets were centrifuged at 600*g* for 2 min and washed twice with ice-cold DPBS.

### Expression Analysis by Semiquantitative RT-PCR

For semi-quantitative RT-PCR total RNA of enterocytes from crypts of proximal and distal colon were isolated using NucleoSpin RNA II columns (Macherey-Nagel, Düren, Germany). Semiquantitative RT-PCR has been described previously (Benedetto et al., 2019b). In brief, total RNA of enterocytes from crypts of proximal and distal colon were isolated using NucleoSpin RNA II columns (Macherey-Nagel, Düren, Germany). Total RNA (1 μg/50 μl reaction) was reverse-transcribed using random primer (Promega, Mannheim, Germany) and M-MLV Reverse Transcriptase RNase H Minus (Promega, Mannheim, Germany). Each RT-PCR reaction contained sense (0.5 μM) and antisense primer (0.5 μM) (Table 1), 0.5 μl cDNA and GoTaq Polymerase (Promega, Mannheim, Germany). After 2 min at 95°C cDNA was amplified (targets 30 cycles, reference Gapdh 25 cycles) for 30 s at 95°C, 30 s at 56°C and 1 min at 72°C. PCR products were visualized by loading on Midori Green Xtra (Nippon Genetics Europe) containing agarose gels and analyzed using ImageJ. Methods for real-time RT-PCR have been reported previously (Schreiber et al., 2010).

**TABLE 1:** Primers used for semiquantitative RT-PCR.

**Gene accession number**	**Primer**	**Size (bp)**
Tmem16a (Ano1)	s: 5′-GTGACAAGACCTGCAGCTAC	406
NM_178642	as: 5′-GCTGCAGCTGTGGAGATTC	
Tmem16b (Ano2)	s: 5′-CCAGAGGAAAGTCGACTATG	544
NM_153589	as: 5′-GGTAGCATTGTCAAAGAAGG	
Tmem16c (Ano3)	s: 5′-TGATAAAAGAAACACATTTGAAAAGAA	611
NM_001081556	as: 5′-GAGGCTGATGCTTGTACCAC	
Tmem16d (Ano4)	s: 5′-TGGCTTCATTTTTGCTGTTCT	555
NM_178773	as: 5′-GAAGAGCATGCCTGTGTACC	
Tmem16e (Ano5)	s: 5′-TCCTGAGGAGGCGTCTTATG	548
NM_177694	as: 5′-CCCAATCTTTTTCTTCCCCTC	
Tmem16f (Ano6)	s: 5′-CATACGAATCTAACCTTATCTGC	520
NM_175344	as: 5′-CATTCTCTGTACAGGAGGTAAC	
Tmem16g (Ano7)	s: 5′-TTGGAATCCGAAATGAGGAG	584
NM_207031	as: 5′-GTGTGCGGAGGTGAAAGTG	
Tmem16h (Ano8)	s: 5′-CTTGGAGGACCAGCCAATC	682
XM_889480	as: 5′-CTTCTTGTAGCCCTCAGCAC	
Tmem16j (Ano9)	s: 5′-CAAGATGTTAAAGGACCAGAAG	487
NM_178381	as: 5′-GAAGATATCATTGGCACTACAG	
Tmem16k (Ano10)	s: 5′-GGACATGAAGCTTTTGCGCC	566
NM_133979	as: 5′-TGGCAAATGCGAGTATGAAC	
Cftr (Abcc7)	s: 5′-GAATCCCCAGCTTATCCACG	544
NM_021050	as: 5′-CTTCACCATCATCTTCCCTAG	
Gapdh	s: 5′-GTATTGGGCGCCTGGTCAC	200
NM_001289726	as: 5′-CTCCTGGAAGATGGTGATGG	

### Western Blotting

Isolation of proteins and Western blotting has been described earlier (Benedetto et al., 2017). The anti-TMEME16A antibody #P49 from Davids Biotechnology (Regensburg, Germany) and the antibody kindly provided by Harfe and Rock (Romanenko et al., 2010) were used at a dilution of 1:500 in 1% NFM/TBST. The anti-ß-actin was from Sigma (Taufkirchen, Germany) was used at a dilution of 1:500 in 1% NFM/PBST.

### Short Circuit Ussing Chamber and Whole Cell Patch Clamp

Stripped colon sections were put into an ice-cold solution of the following composition (in mM): NaCl 140, KCl 5, MgCl_2_.6H_2_0 1, CaCl_2_.2H_2_0 2, HEPES-Tris 10, D-glucose 10, pH 7.4, containing DTT (1 mM) and indomethacin (1 μM). Tissues were mounted in 0.0031 cm^2^ surface area tissue-holders and placed into a short circuited Ussing chamber (Physiologic Instruments, United States). Tissues were bathed in bicarbonate-buffered Ringer solution of the following composition (in mM): NaCl 120, KH_2_PO_4_ 0.4, K_2_HPO_4_.3H_2_0 1.6, D-glucose 5, MgCl_2_.6H_2_0 1, Ca-gluconate.1H_2_0 1.3, NaHCO_3_ 25, and circulated with 95% O_2_-5% CO_2_ gas, pH 7.4; or bicarbonate-free Ringer solution of the following composition (in mM): NaCl 120, KH_2_PO_4_ 0.4, K_2_HPO_4_.3H_2_0 1.6, D-glucose 5, MgCl_2_⋅6H_2_0 1, Ca-gluconate.1H_2_0 1.3, Na-gluconate 25, pH 7.4, and circulated with air. Bath solutions were kept at 37°C during experiments by using a water jacket. The epithelium was voltage-clamped, and short-circuit current (I_sc_) and resistance (R_t_) were measured. Data were acquired and analyzed using Acquire and Analysis (version 2.3) software (Physiologic Instruments). Isolation of colonic crypts and methods for whole cell patch clamp recordings have been described previously (Benedetto et al., 2017).

### Materials and Statistical Analysis

All animal experiments were approved by local authorities and were conducted according to the guidelines of the American Physiological Society and the German law for welfare of animals. All compounds used were of highest available grade of purity. Data are reported as mean ± SEM. Student’s *t*-test (for paired or unpaired samples as appropriate) or ANOVA were used for statistical analysis. A *p*-value < 0.05 was accepted as significant difference.

## Results

### Intestinal Epithelial Expression of Tmem16a Is Detected by Semiquantitative RT-PCR

**FIGURE 1:**
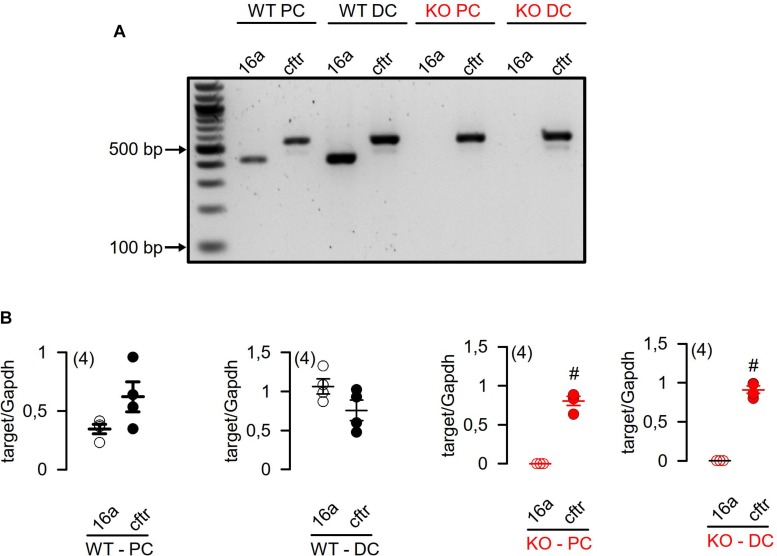
RT-PCR analysis of Tmem16a and Cftr mRNA expression in isolated epithelial cells from proximal and distal colon of WT (Tmem16a^fl/fl^) and KO (Tmem16a^fl/fl^Vil1Cre) mice. **(A)** Expression of Tmem16a (16a) and Cftr mRNA was not different in isolated epithelial cells of proximal (WT PC) and distal colon (WT DC) of WT (Tmem16a^fl/fl^) mice. Knockout of TMEM16a did not affect mRNA expression of Cftr mRNA in isolated epithelial cells from proximal (KO PC) and distal colon (KO DC) of Tmem16a^fl/fl^Vil1Cre mice. **(B)** Quantification of Tmem16a (16a) and Cftr mRNA expression to Glyceraldehyde 3-phosphate dehydrogenase (Gapdh) mRNA expression in isolated epithelial cells of proximal and distal colon. ^#^significant different (*t*-test) (number of measurements).

**FIGURE 2:**
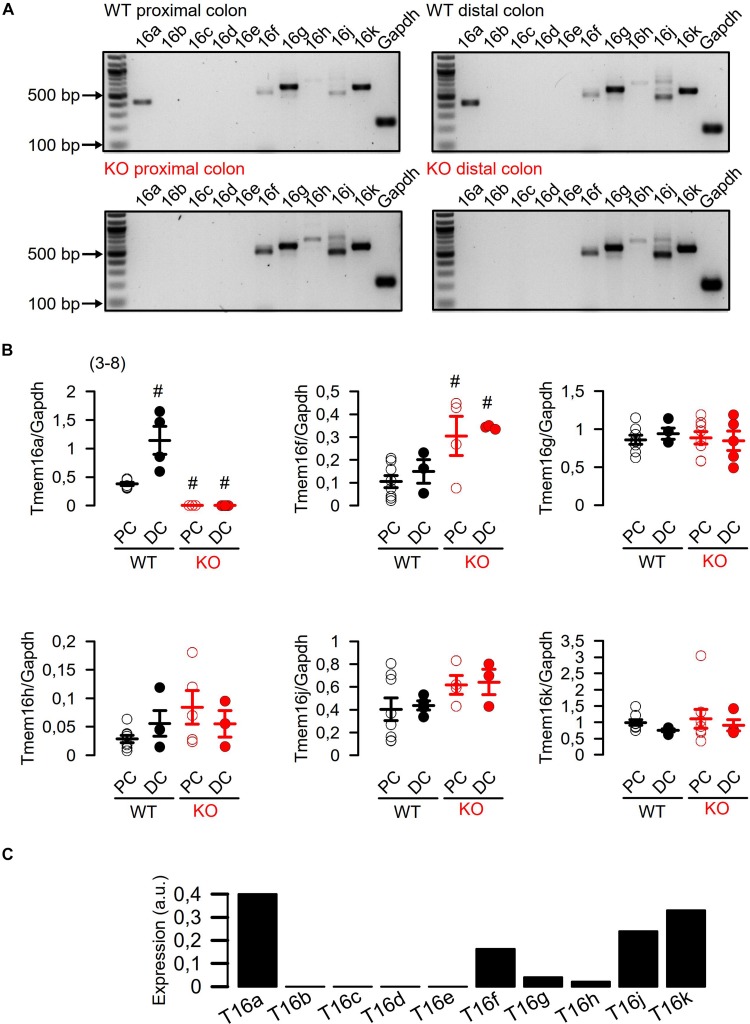
Expression of TMEM16 paralogs in proximal and distal epithelium of WT (Tmem16a^fl/fl^) and KO (Tmem16a^fl/fl^Vil1Cre) mice. **(A)** Expression of Tmem16a to k in isolated epithelial cells from proximal and distal colon of WT (Tmem16a^fl/fl^) and KO (Tmem16a^fl/fl^Vil1Cre) mice. **(B)** Quantification of Tmem16a, f, g, h, j, and k mRNA expression to Glyceraldehyde 3-phosphate dehydrogenase (Gapdh) mRNA expression. **(C)** Real-time RT-PCR of TMEM16a-k in mouse colon. ^#^significant different (ANOVA) (number of measurements).

We analyzed the mRNA expression using semiquantitative RT-PCR. Vega et al. (2019) claimed a very low expression of Tmem16a when compared to “relevant” intestinal ion transport proteins such as NKCC1. We compared expression of Tmem16a with that of cftr, an ion channel that is certainly relevant for murine intestinal ion transport (Bachmann et al., 2003). As shown in Figure 1, there are no significant differences regarding expression of Tmem16a and cftr in murine WT (Tmem16a^fl/fl^) intestinal epithelium, confirming significant levels of Tmem16a-expression in murine large intestine. Similar levels of protein for Tmem16a and cftr has been reported recently (Benedetto et al., 2017). This result was further supported by real-time RT-PCR analysis (Figure 2C; Schreiber et al., 2010). We also analyzed expression of other TMEM16 paralogs in proximal and distal epithelium and excluded a possible compensatory upregulation of other TMEM16 members in knockout (KO; Tmem16a^fl/fl^Vil1Cre) mice (Figures 2A,B). Apart from Tmem16a, expression of Tmem16f, g, h, j, and k was detected in isolated epithelial cells of proximal and distal colon of WT (Tmem16a^fl/fl^) and KO (Tmem16a^fl/fl^Vil1Cre) mice. Interestingly, for Tmem16f an increased expression was found in proximal and distal colon of the KO mice, while similar expression levels were found for Tmem16g, h, j, and k (Figures 2A,B).

**FIGURE 3:**
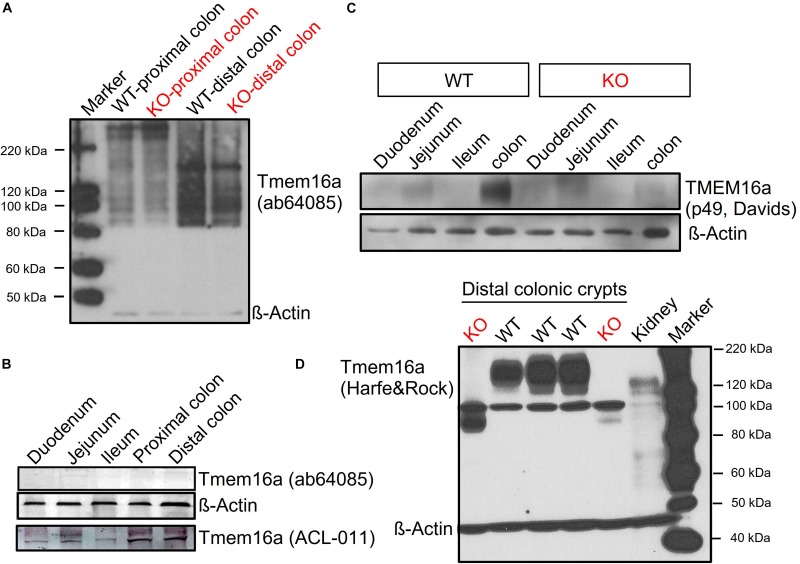
Tmem16a protein is expressed in murine intestinal epithelium. Western blotting of Tmem16a in isolated intestinal crypts. **(A,B)** No bands for Tmem16a could be detected in two different laboratories using the Tmem16a antibody produced by Abcam (Cat#ab64085), while Tmem16a was detected by the antibody from Alamon labs (Cat#ACL-011). **(C)** Detection of Tmem16a by the anti-TMEME16A antibody #P49 from Davids Biotechnology (Regensburg, Germany) in distal colon, but not in small intestine, similar to Benedetto et al. (2019b). **(D)** Using the Tmem16a antibody kindly provided by Harfe and Rock (Romanenko et al., 2010), expression of Tmem16a was found in intestine of three different WT animals, but not in intestine of two different KO animals. Note that this antibody produced a non-specific band at around 100 kDa but was otherwise very clean. Blotting with anti-ß-actin antibody demonstrated equal loading of the lanes. Little Tmem16a was expressed in murine kidney.

Our recent reports demonstrate that the epithelium of distal colon expresses highest levels of Tmem16a, while small intestinal expression of Tmem16a is almost undetectable (Schreiber et al., 2015; Benedetto et al., 2019b). As shown below, this can also be demonstrated by Western blotting (Figure 3C). We conclude that there is a gradual increase in expression of Tmem16a in mouse intestinal epithelium from duodenum/jejunum (no expression), via ileum (little expression), and proximal and distal colon (large expression). These data compare well to our previous analysis.

### Tmem16a Protein Is Expressed in Murine Intestinal Epithelium

Vega et al. (2019) claim lack of expression of Tmem16a protein, which contradicts the findings of 14 previous reports (outlined in section “Introduction”). We examined the commercially available antibody (Abcam Cat#ab64085) used in the paper by Vega et al. (2019). In two independent laboratories (Department of Physiology at University of Regensburg, Germany; National Institute of Cholera and Enteric Disease, Kolkata, India), this antibody was unable to detect expression of Tmem16a in murine colon using Western blotting (Figures 3A,B). In sharp contrast, the TMEM16a antibodies ACL-011, p49, and the antibody provided by Harfe and Rock (Romanenko et al., 2010) demonstrated clear expression of TMEM16A in WT intestinal epithelium (isolated crypts), which was absent in KO intestine (Figures 3B–D). The uncropped full length blot in Figure 3D also demonstrates the low level expression of Tmem16a in murine kidney (Faria et al., 2014).

### Reduced Colonic Ion Transport in Tmem16a Knockout Mice

We previously reported attenuated ion transport in colonic epithelia from mice with intestinal epithelial knockout of Tmem16a (Tmem16a^fl/fl^Vil1-Cre) (Benedetto et al., 2017). Short circuit currents activated by carbachol (Ca^2+^ dependent stimulation) and by IBMX/forskolin (cAMP dependent stimulation) were clearly attenuated in intestine from Tmem16a^fl/fl^Vil1-Cre mice. These experiments were performed under open circuit conditions and in the absence of CO_2_/bic. Here we used short circuit conditions and performed the experiments in the presence of CO_2_/bic in a commercial non-perfused bubble lift Ussing chamber (Physiological Instruments, United States). We compared ion transport between WT (Tmem16a^fl/fl^; *n* = 5 animals) and KO (Tmem16a^fl/fl^Vil1-Cre; *n* = 7 animals). Basal I_sc_ were similar in proximal colon [54.2 ± 13.5 (WT; *n* = 14) and 66.1 ± 20.4 (KO; *n* = 6) μA/cm^2^], but were larger in WT distal colon [81.4 ± 13.0 (WT; *n* = 13) when compared to KO 31.7 ± 21.4 (*n* = 6) μA/cm^2^].

**FIGURE 4:**
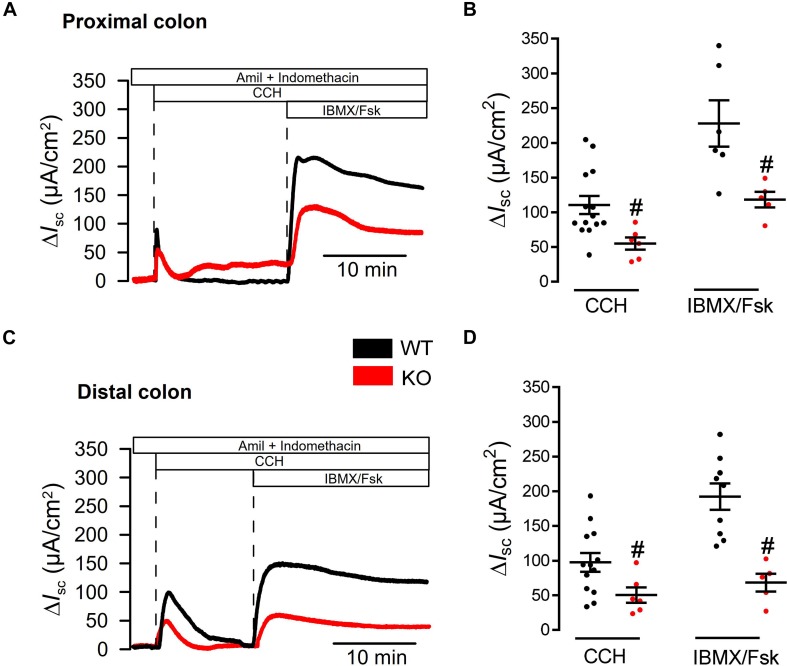
Defective ion transport in colon of Tmem16a^fl/fl^1VilCre mice. **(A,C)** Original recordings for CCH (100 μM) and IBMX/Fsk (100 μM/2 μM) activated short circuit currents in WT (Tmem16a^fl/fl^) and KO (Tmem16a^fl/fl^1VilCre) colon, after subtraction of baseline currents measured before stimulation. Transport was assessed in proximal and distal colon. **(B,D)** Scatter blot summarizing individual experiments. Activation of I_sc_ by CCH and IBMX/Fsk were significantly attenuated in intestine of KO mice, when compared to WT littermates. Mean ± SEM. Number of animals 5–7. ^#^indicates significant difference (unpaired *t*-test).

We perfused tissues with amiloride and indomethacin (both 10 μM) to inhibit electrogenic Na^+^ absorption and endogenous cftr activity. Then tissues were stimulated with basolateral carbachol (CCH, 100 μM) and subsequently with luminal IBMX and forskolin (IBMX/Fsk, 100 and 2 μM). While CCH induced an only transient I_sc_, IBMX/Fsk activated a more steady ion transport as expected from earlier studies (Mall et al., 1998; Benedetto et al., 2017). Both, CCH and IBMX/Fsk activated transport were attenuated in proximal colon of KO animals (Figures 4A,B). Similar results were obtained when ion transport was compared between distal colon from WT and KO animals (Figures 4C,D). Thus, short circuit measurements performed under short circuit conditions in the presence of CO_2_/bic, correspond well to results reported earlier under open circuit conditions and in the absence of CO_2_/bic (Benedetto et al., 2017).

**FIGURE 5:**
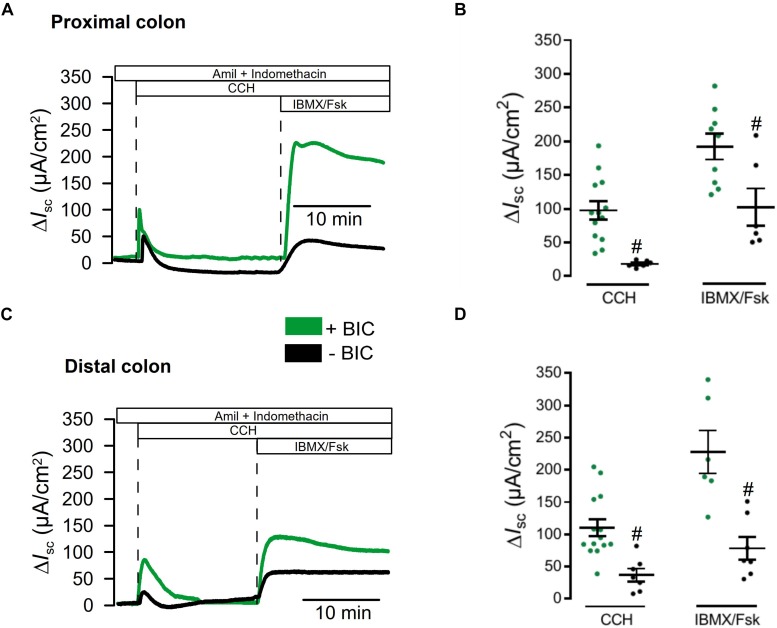
Impact of CO_2_ and bicarbonate on ion transport in large intestine. **(A,C)** Original recordings for CCH (100 μM) and IBMX/Fsk (100 μM/2 μM) activated short circuit currents, after subtraction of baseline currents measured before stimulation. Transport was assessed in proximal and distal colon in the presence of absence of 5%CO_2_/25 mM bicarbonate. **(B,D)** Scatter blot summarizing individual experiments. Activation of I_sc_ by CCH and IBMX/Fsk was significantly attenuated in the absence of CO_2_/bicarbonate. Mean ± SEM. Number of animals 5–7. ^#^indicates significant difference (unpaired *t*-test).

We had the impression that ion transport measured in the presence of CO_2_/bic was generally larger than in earlier measurements in the absence of CO_2_/bic (Benedetto et al., 2017). We therefore compared the effects of CCH and IBMX/Fsk in the absence and presence of CO_2_/bic. Basal I_sc_ were larger in the presence of CO_2_/bic in proximal colon 54.2 ± 13.5 (+CO_2_/bic; *n* = 14) vs. 27.51 ± 21.8 μA/cm^2^ (−CO_2_/bic; *n* = 7) and distal colon 81.4 ± 13.0 (+CO_2_; *n* = 13) vs. 12.2 ± 5.2 μA/cm^2^ (−CO_2_/bic; *n* = 6). Moreover, Ca^2+^- and cAMP-activated ion transport was clearly enhanced in the presence of CO_2_/bic (Figure 5). These data indicate that a substantial portion of both Ca^2+^-activated and cAMP-regulated transport is indeed supported by bicarbonate and Cl^–^ uptake, most likely through NBC1-mediated transport (Seidler et al., 2001).

**FIGURE 6:**
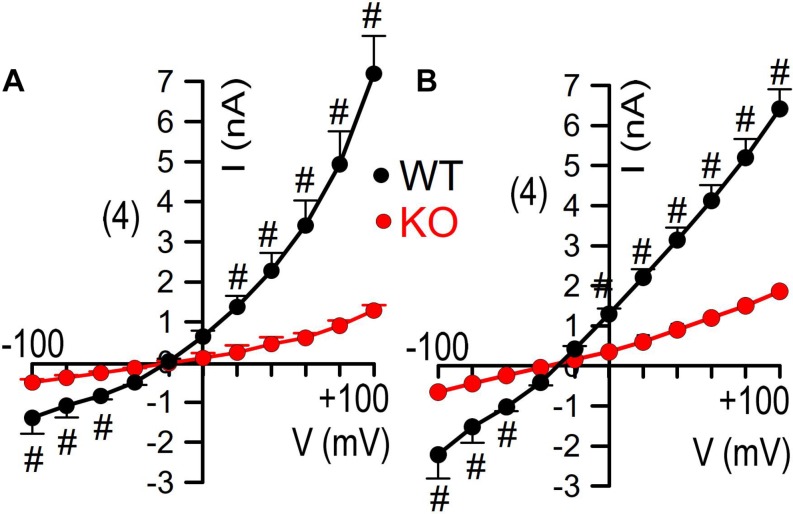
Inhibited whole cell currents in isolated colonocytes from KO mice. Current/voltage relationships for whole cell currents activated by CCH [100 μM; **(A)**] and IBMX/forskolin [100/2 μM; **(B)**] in freshly isolated colonocytes obtained from WT and KO animals Mean ± SEM. Number of animals 3. ^#^indicates significant difference compared to KO (unpaired *t*-test).

**FIGURE 7:**
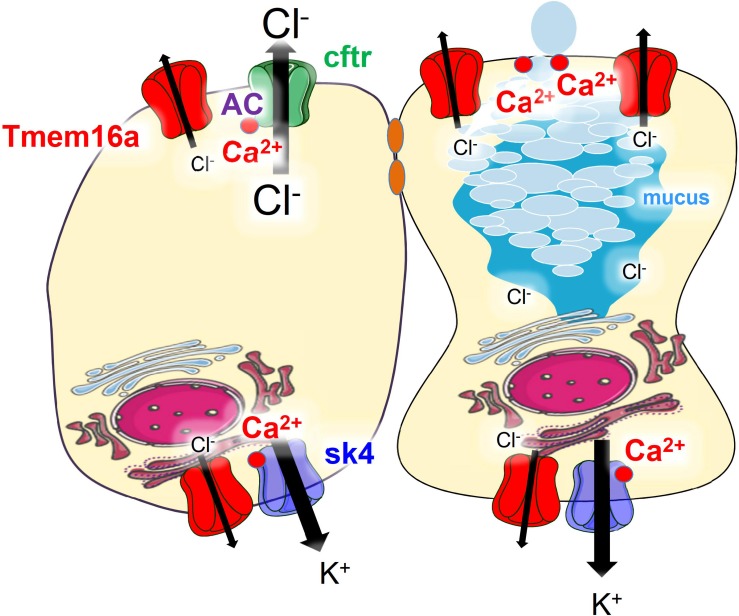
Model of the predicted role of Tmem16a in murine colonic epithelium. Tmem16a is expressed in both murine intestinal epithelial and goblet cells, predominantly in the basolateral membrane. In adult normal murine large intestine, CFTR is the only apical Cl^–^ channel and Tmem16a does not directly serve as luminal channel for secretion of Cl^–^. Through mechanisms outlined in previous reports (Schreiber et al., 2015; Cabrita et al., 2017), Tmem16a enables compartmentalized Ca^2+^ signals close to apical and basolateral membranes, which support activation of CFTR (apical) and Sk4 Ca^2+^-activated K^+^ channels (basolateral) in fluid secreting cells. In goblet cells it supports apical exocytosis of mucus-filled granules (Benedetto et al., 2019a).

Finally, we reexamined whole cell currents activated by either Ca^2+^ or by cAMP in freshly isolated colonic crypts. Current/voltage relationships indicated strongly reduced whole cell currents in colonic crypt cells obtained from KO animals (Figure 6). Taken together reanalysis of TMEM16a expression and functional analysis provided results that corresponded well with our previous observations. We therefore conclude that TMEM16A is important for electrogenic Cl^–^ secretion and may facilitate mucus release by goblet cells of murine intestine (Figure 7).

## Discussion

The present and previous data indicate expression of Tmem16a in murine colonic epithelium. Tmem16a controls Ca^2+^ and cAMP-activated Cl^–^ transport (Schreiber et al., 2015; Benedetto et al., 2017; Lee et al., 2019). In contrast, Vega et al. (2019) reported lack of expression of Tmem16a in murine intestinal epithelium, and did not detect a difference in Ca^2+^- and cAMP-activated transport between WT and Tmem16a knockout animals. We noticed some inconsistencies in their transcript analysis, such as different length of PCR products present in small and large intestine (Supplement S1). Although we did not notice such differences in our previous analysis, they might be due to alternatively spliced Tmem16a products in ileum and colon (Ferrera et al., 2009).

Transcript and proteome analysis in Vega et al. (2019) detected very low levels of Tmem16a in ileum, but showed significantly higher expression of Tmem16a in distal colon, a result that confirms our previous results (Schreiber et al., 2015). Vega et al. (2019) argue that even in the distal colon the level of Tmem16a expression is low, when compared to the cotransporter Nkcc1, implying a minimal or no role at all of Tmem16a for ion transport. This conclusion, however, is not valid because of the following reasons: (i) Epithelial ion channels are generally expressed at lower levels compared to cotransporters or pumps. While ion channels transport up to 10^7^ ions/s, a transporter can only handle maybe up to 10^3^ ions/s (Hille, 1978). Thus thousands of transporters are required to keep up with the transport enabled by a few ion channels. (ii) Compared to murine large intestine, murine airways express even lower levels of Tmem16a. Yet, a significant role of Tmem16a for murine airway ion transport is not questioned (Benedetto et al., 2017). (iii) Our proposed concept for the role of Tmem16a in adult murine intestinal ion transport is not that of an apical secretory Cl^–^ channel (Schreiber et al., 2015; Benedetto et al., 2017). In other words, cftr is most likely the only luminal exit pathway for Cl^–^ ions in the adult murine and human colon. This is because the absence of cftr (CF knockout), complete inactivation of cftr (indomethacin), and complete blockage of cftr (inhibitors) abolish not only cAMP-activated Cl^–^ secretion, but also Ca^2+^ activated Cl^–^ secretion. We were the first demonstrating that Ca^2+^ dependent Cl^–^ secretion in the human colon occurs through apical cftr, and that no other (Ca^2+^ activated) Cl^–^ channel is present in the apical membrane of human colorectum (Mall et al., 1998; Hirtz et al., 2004). Some evidence exists for enhanced apical Tmem16a in pups and under pathological conditions such as rotavirus expression (Ball et al., 1996; Ousingsawat et al., 2011). Accordingly, the Verkman team demonstrated inhibition of rotavirus-induced diarrhea by TMEM16-inhibitors (Ko et al., 2014). In the adult colon we and others localized Tmem16a predominantly to the basolateral side of the epithelium, although it can be also found in the apical membrane of goblet cells and enterocytes (He et al., 2011; Schreiber et al., 2015; Benedetto et al., 2019a). Finally, in the present paper we again found comparable levels of expression for Tmem16a and cftr in murine colon (Figure 1; Benedetto et al., 2017).

We may speculate that Vega et al. (2019) did not detect Tmem16a protein in mouse intestine, because they used the antibody ab64085. This antibody failed to detect Tmem16a protein in two different laboratories, while expression was found using three additional antibodies. Using ab64085, Vega et al. (2019) showed a band Tmem16a band of 114 kDa for smooth muscle. Vega et al. (2019) did not find a difference in Ca^2+^ activated Cl^–^ transport in WT and KO intestine, although previous work detected a clear impact of Tmem16a on both Ca^2+^ activated as well as cAMP-regulated Cl^–^ secretion (Hoque et al., 2012; Schreiber et al., 2015; Benedetto et al., 2017; Lee et al., 2019; Vega et al., 2019). The recordings in Vega et al. (2019) were performed under open circuit conditions, but recordings are shown as carbachol-induced short circuit currents, which is not further explained. If the recordings reflect calculated amounts of activated equivalent short circuits (as indicated), it is unclear why the currents don’t start at 0 μA/cm^2^ (before stimulation) and why equivalent I_sc_ are negative. Along with other problems such as variability and very short duration of the recordings this is difficult to interpret. We reanalyzed ion transport, this time in a short-circuited Ussing chamber and in the presence of CO_2_/bic. Again, the results demonstrate a clear impact on Ca^2+^- and cAMP-activated Cl^–^ transport (Figure 4; Schreiber et al., 2015; Benedetto et al., 2017).

Vega et al. (2019) did not find a difference in mucus “architecture.” However, they provide only single stainings without presenting any statistical analysis of intestinal luminal and intracellular mucus, respectively. Such an extensive analysis was done previously, which uncovered a role of Tmem16a for mucus release (Benedetto et al., 2019a). Accordingly, a concept was proposed for the role of Tmem16a in purinergic mucus release (Figure 7). We also demonstrate inhibition of ATP-induced intestinal mucus secretion by acute application of the Tmem16a-blocker niclosamide (Cabrita et al., 2019; Miner et al., 2019). Preliminary experiments also demonstrate reduced intestinal mucus load upon 7 days treatment of mice with niclosamide, which did not compromise intestinal function and survival (not shown). The uncompromised cholinergic mucus secretion in the KO animals provides an explanation for this finding (Benedetto et al., 2019a; Cabrita et al., 2019).

## Conclusion

Tmem16a is expressed in murine colon at levels comparable to other ion channels such as cftr or sk4 (Preston et al., 2010). Tmem16a controls Ca^2+^-regulated and cAMP-dependent Cl^–^ secretion. Tmem16a/f are relevant for intestinal mucus secretion and mucus production.

## Data Availability Statement

The datasets generated for this study are available on request to the corresponding author.

## Ethics Statement

The animal study was reviewed and approved by the Government of Unterfranken/Würzburg (AZ: 55.2-2532-2-328).

## Author Contributions

KK, RS, and KH designed the study. RC, PW, IC, RB, TS, and RS carried out the experiments and analyzed the data. KK made the figures. KK, KH, and RS drafted the manuscript. All authors approved the final version of the manuscript.

## Conflict of Interest

The authors declare that the research was conducted in the absence of any commercial or financial relationships that could be construed as a potential conflict of interest.

## Supplementary Material

SUPPLEMENT S1 Additional comments to the report by Vega et al. (2019). (A) Suboptimal annealing temperatures may affect results from RT-PCR. (B) RT-PCR providing unclear results genotyping results.
