# Supplementary material for: Control of Ion Transport by Tmem16a Expressed in Murine Intestine
Source: Front Physiol. 2019 Oct 4;10:1262. doi: 10.3389/fphys.2019.01262 (PMC6797858; doi:10.3389/fphys.2019.01262)
Supplement: SUPPLEMENT S1 — Additional comments to the report by Vega et al. (2019). (A) Suboptimal annealing temperatures may affect results from RT-PCR. (B) RT-PCR providing unclear results genotyping results. [file Data_Sheet_1.PDF]

Supplement 1

High annealing temperatures for primes (TMEM16A) used in Vega et al [1].

Calculated (GC+AT) Tm	Tm used in [1].
Tmem16A sense 60°C	used 63°C
Tmem16A antisense 58°C	used 63°C
Tmem16A Exon 6-16 sense 58°C	used 61.3°C
Tmem16A Exon 6-16 antisense 60°C	used 61.3°C

Unclear results for genotyping in Vega et al [1]

Vega et al used a similar Vil1Cre mouse and the same floxed TMEM16A mouse we used in our previous studies [2; 3]. Crossbreeding of these mice leads to deletion of exon 12 (159 bp). Animals were genotyped by RT-PCR with one primer located in exon 12, which provides unequivocal results [2; 3]. Vega et al put both primers into flanking sequences and analyzed the length of the PCR product. This can be challenging, particularly when differences in product size are small. Deletion of exon 12 causes a shortening of the PCR products by 159 bp. However, bands of variable sizes are shown in Fig. 2A for TMEM16A$^{fl/fl}$Vil1Cre in ileum and colon [1]. To confirm a specific deletion of exon 12 from the *Tmem16a* gene, mRNA isolated from epithelium and smooth muscle was retro-transcribed and screened by PCR with primers harboring exons 6 to 16. Wild type Ileum presented a PCR product of ~800 bp (Fig. 2A), which according to our analysis should be 700 bp. The PCR product of WT ileum should be 762 bp, while that of KO ileum should be around 600 bp. After deletion of exon 12 (which should have a length of 159 bp instead of 83 bp) a band between 540-600 bp is expected, but instead a PCR product of 700-750 bp is shown (Fig. 2A) [1]. For proximal colon a ~750-800 bp band is shown, but a PCR product of 700 bp is expected. For *Tmem16*fl/fl/*Villin*cre/- proximal colon, a ~600 bp band is shown but a 540 bp band.

References

[1] G. Vega, A. Güequén, M.E. Johansson, L. Arike, B. Martínez-Abad, P. Scudieri, N. Pedemonte, P. Millar-Büchner, A.R. Philp, L.J. Galietta, G.C. Hansson, and C.A. Flores, Normal calcium-activated anion secretion in a mouse selectively lacking TMEM16A in intestinal epithelium. . Front Physiol 10 (2019) 694.

[2] R. Schreiber, D. Faria, B.V. Skryabin, J.R. Rock, and K. Kunzelmann, Anoctamins support calcium-dependent chloride secretion by facilitating calcium signaling in adult mouse intestine. Pflügers Arch 467 (2015) 1203-1213.

[3] R. Benedetto, J. Ousingsawat, P. Wanitchakool, Y. Zhang, M.J. Holtzman, M. Amaral, J.R. Rock, R. Schreiber, and K. Kunzelmann, Epithelial Chloride Transport by CFTR Requires TMEM16A. Scientific Reports 7 (2017) 12397.
